# Chemotherapy Promotes Release of Exosomes Which Upregulate Cholesterol Synthesis and Chemoresistance in AML Blasts

**DOI:** 10.33696/haematology.2.026

**Published:** 2021

**Authors:** Chang-Sook Hong, Michael Boyiadzis, Theresa L. Whiteside

**Affiliations:** 1 Department of Pathology, University of Pittsburgh and UPMC Hillman Cancer Center, Pittsburgh, PA 15213, USA; 2 Department of Hematology, University of Pittsburgh and UPMC Hillman Cancer Center, Pittsburgh, PA 15213, USA; 3 Department of Immunology, University of Pittsburgh and UPMC Hillman Cancer Center, Pittsburgh, PA 15213, USA

Extracellular vesicles (EVs) are emerging as a key mediator of intercellular communication as well as a major mechanism of functional reprogramming of cells in disease [1-2]. All cells produce EVs, which freely circulate and are found in all body fluids. EVs are heterogenous, consisting of subsets of vesicles with different sizes, distinct origins, and various functions (Figure 1). They mediate a broad variety of biological events ranging from cellular activation, inflammation, blood coagulation, angiogenesis, cellular transport, and others. Among these vesicles, a subset of small EVs (30-150 nm in diameter) originating from multivesicular bodies (MVBs) in parent cells and referred to as small extracellular vesicles (sEVs) carry proteomic, genomic and functional signatures that resemble those of parent cells and are, therefore, taken as surrogates of parent cells. In cancer, tumor-derived exosomes (TEX) reflect characteristics of tumor cells and are considered candidates for “liquid tumor biopsy” [3]. Emerging evidence shows that TEX are a major sEV subset in plasma of patients with cancer, including hematologic malignancies [4].

Plasma of AML patients contains a variety of EVs, some derived from leukemic blasts and others from non-malignant blood and tissue cells. In acute myeloid leukemia (AML), sEVs accumulate in plasma of patients, and total exosome protein (TEP) levels at AML diagnosis, prior to any primary chemotherapy, are significantly elevated relative to those seen in healthy donor’s (HD’s) plasma [5]. The protein profile of these sEV is enriched in leukemia-associated antigens (LAAs), such as CD34, CD123, CD96, CLL-1, suggesting these are leukemia blast-derived sEVs [6]. We were interested in determining whether plasma levels of these LAA+ sEVs change during standard of care (SOC) chemotherapy (CT) in patients with AML. To isolate sEV from plasma of patients, we used size exclusion chromatography (SEC) preceded by differential centrifugation and ultrafiltration. This method of isolation utilizes 1 ml of plasma, which yields 10^9^ to 10^10^ sEVs in fraction #4 [6]. We observed that TEP levels of sEVs isolated from plasma of patients in complete remission (CR) after SOC CT remained highly elevated and were similar to the levels at AML diagnosis. Further, these TEP levels remained elevated in patients who relapsed. These observations prompted us to ask whether elevated TEP levels in AML plasma after first line SOC therapy could reflect excessive packaging of the drug into sEVs and their release by leukemic blasts resistant to CT. It is well known that resistance of leukemic blasts to CT is a major cause of death, and recent data have intimated that sEVs might contribute to or mediate chemoresistance [7].

In the recently published article entitled “Increased small extracellular vesicle secretion after chemotherapy via upregulation of cholesterol metabolism in acute myeloid leukemia” [8], we reported that exosomes play a critical role in AML progression by establishing and driving a vicious cycle of chemoresistance in leukemic blasts. The mechanisms involved in exosome-mediated chemoresistance of AML blasts to CT are largely unknown, but our data suggest that it may be related to the excessive sEV release by AML blasts. At the same time, we and others have observed that AML blasts exposed to CT contain high cholesterol levels [9-10]. Lipids, including cholesterol, have been linked to sEV packaging and secretion [11]. Further, CT or ionizing radiation as well as other stress inducing conditions increase cholesterol levels in cancer cells [12-13]. Therefore, it is possible that an excess of sEV observed in AML plasma after CT is related to cholesterol accumulation in AML blasts. We investigated the potential relationship between CT, cholesterol accumulation by cells and an excessive sEV release using AML cell lines. We focused on an enzyme called 3-hydroxy-3-methylglutaryl coenzyme A reductase (HMGCR) which regulates cholesterol synthesis in the mevalonate pathway and which is inhibited by statins, including Simvastatin. In a series of *in vitro* experiments with AML cell lines, we showed that Cytarabine or Decitabine, CTs commonly used for AML, significantly enhanced expression of HMGCR in CT-treated cells and increased cholesterol levels as well as sEV release from these cells. Simvastatin inhibited HMGCR activity, i.e., cholesterol synthesis, as well as sEV release from CT-treated AML cells. Blocking of HMGCR gene translation with siRNA reduced sEV production by these cells. These experiments showed that CTs significantly upregulated cholesterol synthesis and sEV production/release by AML. In cell cultures, AML cells co-incubated with HMGCR (+) sEVs expressed higher levels of HMGCR and proliferate better than control cells.

Importantly, sEVs isolated from plasma of CT-treated AML patients by ultrafiltration and SEC were found to be LAA (+) and HMGCR (+). Further, these HMGCR (+) sEVs from AML plasma were rapidly internalized by AML cells or non-malignant recipient cells. Moreover, uptake of these HMGCR (+) sEVs led to increased cholesterol synthesis as well as proliferation of AML cells and further enhanced release of HMGCR (+) sEVs by the rapidly expanding AML cells. In other words, blast-derived sEVs in plasma of AML patients treated with CT induced chemoresistance by up-regulating HMGCR activity, cholesterol production and proliferation in cultured naïve AML cells and also upregulated a massive release of HMGCR (+) sEVs into extracellular space. The autocrine mechanism initiated and mediated by blast-derived sEVs sets up a cycle of events that is mechanistically driven by the upregulation of cholesterol metabolism and the release of HMGCR (+) sEVs. This sEV-driven mechanism not only allows AML cells to avoid death, presumably by packaging drugs into vesicles, but it promotes expansion of cholesterol-enriched chemoresistant AML cells which, in turn, promote further release of HMGCR (+) sEVs. This accommodating cross-talk between cancer cells and sEV secretion works against efficacy of chemotherapy and establishes a vicious cycle of chemoresistance. Further, since the blast-derived sEVs in plasma of AML patients have been shown to carry immunosuppressive molecules such as TGFb-1, FasL, PD-L1 [6], their elevated levels in plasma contribute to inhibition of anti-leukemia immune responses, additionally promoting cancer progression. Our experiments showed that blast-derived HMGCR+ sEVs are the central and key element in this vicious cycle of chemoresistance in AML.

Chemoresistance induced by sEV is not limited to sEVs produced by tumor cells. While cancer cell-derived sEVs deliver molecules derived from parental cancer cells, sEVs released from non-malignant cells in the context of CT might also play a significant role in cancer promotion and chemoresistance. We found that sEVs isolated from post-CT AML plasma which contained no detectable leukemia blasts had high levels of HMGCR and other immunosuppressive molecules. *Ex vivo* studies with non-malignant peripheral blood mononuclear cells (PBMC) showed that CT treatments similarly enhanced sEV secretion and that the released sEVs carried elevated levels of enzymatically active HMGCR which was blocked by statins. This finding implies that HMGCR (+) sEVs in the peripheral circulation might impede cholesterol level reducing efficacy of statins. In such case, blocking of the sEV release and/or the sEV uptake by recipient cells or removal of circulating sEVs represent potentially promising strategies for enhancing therapeutic efficacy of statins and lowering chemoresistance in patients with cancer treated with CT.

In the *in vitro* experiments, treatment with Simvastatin, the HMGCR inhibitor, blocked CT-induced enhancement of sEV release from AML cells. This suggests that the use of statins in combination with SOC CT could reduce cholesterol levels in cells and in sEVs thus reducing the overall sEV burden and potentially improving the efficacy of chemotherapy. Indeed, various studies, including Phase I and II clinical trials, have reported on the beneficial effects of statins in cancer treatments [14]. In relapsed AML cases, treatments with Pravastatin in conjunction with Idarubicin and Cytarabine significantly improved complete remission rates [15]. While these studies have not been focused on the role sEVs might play in the treatment efficacy, it seems reasonable to suggest that the beneficial effects of statins in cancer patients could be enhanced by lowering the harmful burden of HMGCR+ sEV in the blood stream, thereby sensitizing cancer cells to chemotherapy. Alternatively, sEVs might uptake circulating statins, carry statins to cancer cells and by lowering cellular cholesterol levels increase tumor cell sensitivity to chemotherapy. This study suggests that the use of these well-tolerated, widely used cholesterol level-reducing drugs could be a potential solution for blocking detrimental effects of tumor-derived HMGCR +sEVs in hematological and other malignancies.

## Figures and Tables

**Figure 1:**
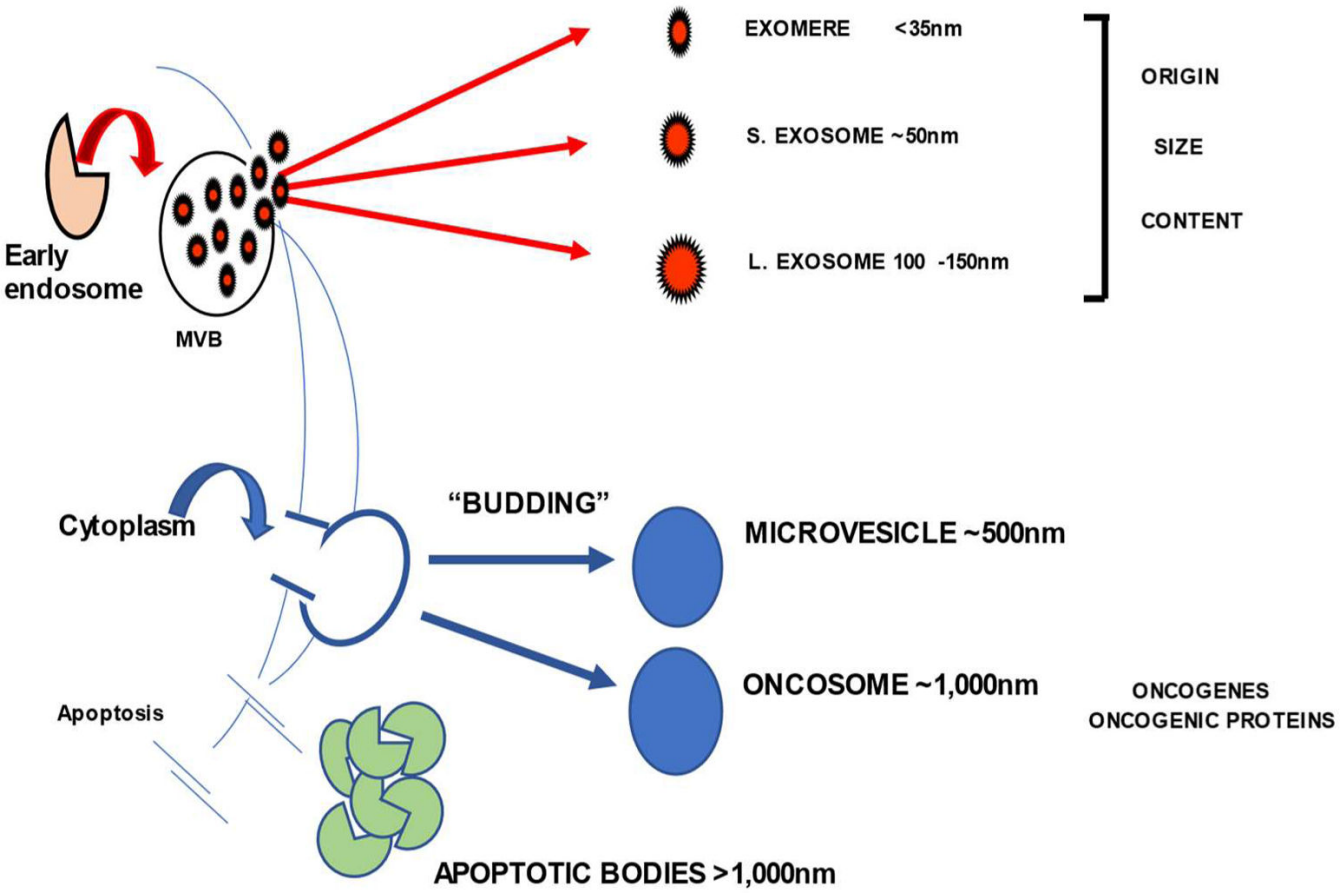
Different types and origins of extracellular vesicles released from cancer cells.
